# Linkages between Respiratory Symptoms in Women and Biofuel Use: Regional Case Study of Rajasthan, India

**DOI:** 10.3390/ijerph16193594

**Published:** 2019-09-25

**Authors:** Priti Parikh, Corina Shika Kwami, Vivekanand Vivekanand, Kunwar Paritosh, Monica Lakhanpaul

**Affiliations:** 1 Civil, Environment and Geomatic Engineering, University College London, London WC1E 6DE, UK; 2 Centre for Energy and Environment, Malaviya National Institute of Technology, Jaipur, Rajasthan 302017, India; 3 Great Ormond Street Institute of Child Health, University College London, London WC1E 6DE, UK; 4 The Whittington Health NHS Trust, The Whittington Hospital, Magdala Avenue, London N19 5NF, UK

**Keywords:** biofuel, women, cooking, Ujjawala, Rajasthan, respiratory infection

## Abstract

Women in low and middle-income countries predominantly use biofuel for cooking, resulting in potential adverse health outcomes. In India, it is estimated that about 40% of total primary energy consumption is in the domestic sector with biofuels alone accounting for about 75% of domestic energy consumption. This study assesses linkages between wood consumption and perceptions of women’s health, combining results from a rapid assessment of eight rural districts in Rajasthan with a regression analysis of data from Rajasthan State (sample size 41,965 women) from the Demographic and Health Survey 7 dataset (2015–2016). The results of the rapid survey indicate that women who cook with biofuels perceive adverse health outcomes. Educational level, income, and age have an impact on fuel consumption and clean fuel purchased. The regression model drawing upon data on women at a regional level in Rajasthan yielded significant results suggesting a strong association between fuel type and symptoms of respiratory infection controlling for age and education. This research is timely as it provides valuable evidence for India’s Ujjawala Scheme which has the mandate of providing LPG connections to women from below the poverty line.

## 1. Introduction

### 1.1. Background on the Link between Cooking Fuel and Respiratory Symptoms Globally

The health outcomes of wood consumption are well established at a global level [1,2] with greater adverse effects experienced by women as they are exposed to health hazards since they often collect, transport, and use wood for cooking [3]. Children also get adversely affected as they are often carried on their mothers’ backs or laps during cooking or are in proximity of cooking stoves when mothers cook [4,5]. As an abundant and widespread used cooking fuel, biomass fuel accounts for a large share of energy consumption in the world. This is particularly acute as “energy access is far from universal—with 1.3 billion people lacking access to electricity and 2.7 billion with no access to modern and health forms of cooking” [6]. Especially in developing countries, low income, population growth, and the rising prices of non-biomass fuel strengthen the trend for using biomass fuel [7] These influencing factors indicate that biomass fuel will continue to be used in developing countries for many decades with biomass potentially accounting for 60% of total final renewable energy used by 2030 [8].

In India, it is estimated that about 40% of total primary energy consumption is in the domestic sector out of which biomass fuels account for about 75% of domestic energy consumption [9]. With population growth, the trend of consumption of biomass fuels has steadily increased. The Government of India, therefore, created a scheme in 2016 which aims to provide LPG connections to women from below the poverty line (BPL) [10] which has received a lot of attention and cautiously positive feedback from communities [2]. 

In all of the household activities, cooking makes the most significant contribution to indoor air pollution and adversely affects women [11]. A study exploring 10,000+ households across 3 states in northern India found that women experience the adverse effects of smoke due to the use of biofuels. At a household level, it is estimated that women spend about 50 hours per month for fuel-wood collection and transportation [12]. In particular, rural households use unprocessed biomass fuels such as wood, twigs, leaves, etc. for cooking in open fireplaces or in non-airtight stoves, which release large quantities of air pollutants including particulate matter and carbon di/monoxides [13]. In a global meta-analysis of 51 studies exploring associations between domestic use of solid biomass fuels and chronic bronchitis in women, it was found that chronic obstructive pulmonary disease (COPD) was higher among women exposed to solid biomass fuel (odds ratio 2.40, 95% CI 1.47–3.93) [14].

This paper’s wider aim is to explore linkages between the use of wood for cooking and health outcomes for women, thereby providing an evidence base at regional and district level for schemes such as Ujjawala which has the goal of improving access to clean cooking fuels with the potential of improving health and reducing indoor air pollution (IAP). 

### 1.2. Problem Statement: Biofuel Use and Respiratory Symptoms in Rajasthan 

Women exposed to biofuel smoke suffer more from respiratory illnesses and have decreased pulmonary functions compared with women exposed to kerosene or LPG smoke as identified by Dutt et al. in Pondicherry, India [15]. The study reported that among 1117 young girls and women aged between 15 and 60 years using biofuels experienced more respiratory symptoms (23%) than those using kerosene [15]. A national study published by the Institute for Health Metrics and Evaluation (IHME) found that in the state of Rajasthan chronic obstructive pulmonary disease (COPD) mortality rate is leading among states in 2016, and deaths from respiratory diseases were the second highest. In 1990, the number of people who died from COPD in Rajasthan accounted for 3.4% of the total number of deaths from respiratory diseases, and this figure increased to 7% in 2016 [16]. The study also found that the proportional contributions of household air pollution and second-hand smoke to COPD disability-adjusted life years (DALYs) were greater in women than in men.

With the burden of respiratory disease increasingly understood as associated with exposure to smoke, there is also growing attention to alternatives to biofuel such as LPG and other forms of clean fuel and new stove types. The uptake of these new alternatives was reported to be highly correlated with marketing tactics and previous stove use [17]. There is also strong evidence that level of wealth is highly correlated with fuel choice and other behavioral aspects of cooking fuel choice, effort to collect wood, and sourcing and cooking practices [18,19] and exposure to domestic cooking fuels produced a significant amount of respiratory morbidity [3,20,21]. Researchers also shared the view of income as a major determinant of fuel use, however, in order to capture microtrends, a range of personal and contextual variables should be included in order to not overemphasize the income [22]. Gender issues have also been identified as associated with the disproportionate level of women experiencing symptoms. 

**Figure 1:**
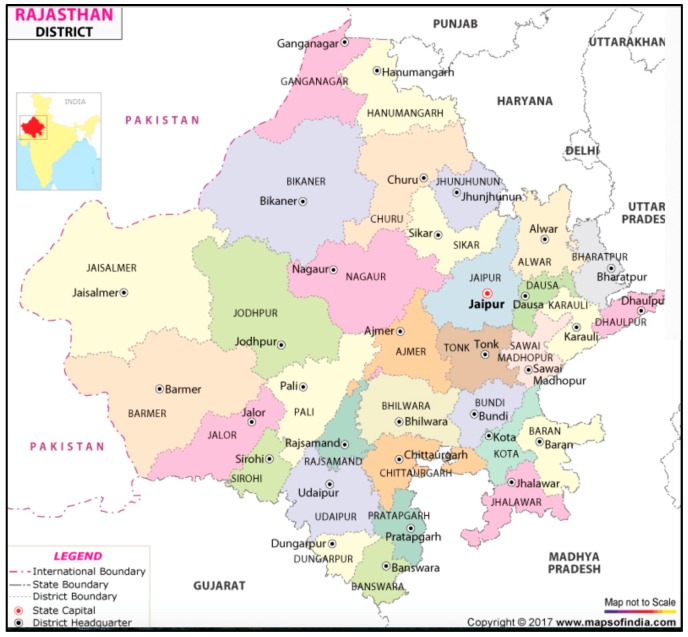
Regional map of Rajasthan and location of Jaipur (Maps of India, 2018).

There are contextual and demographic aspects of Rajasthan that should be taken into account particularly in settings where the health burden is highest and where there is potential to affect change toward clean fuels. Rajasthan is a larger state in India and has been regarded as one of the poorest states in India (Figure 1). The state is divided into 33 districts, of which Jaipur is the administrative center and the largest city. In 2011, the population of Jaipur district reached 6.62 million, of which 3.1 million were urban residents, which meant that Jaipur had become one of the most populous cities in India with widespread unemployment and poverty [23]. 

Rajasthan faces an enormous challenge with regards to the obtained clean energy for rural areas. Price, affordability, and availability of fuel are among the main determinant of fuel use. The households that typically rely on the cheapest available fuels, which are highly associated with negative impact on health and safety, are the most vulnerable who often live in poorly ventilated, overcrowded dwellings. This is an example of the intersection of the Sustainable Development Goals (SDGs) 1 (Poverty), 3 (Health), 7 (Clean Energy), and 11 (Sustainable Cities) which offers an opportunity for integration and exploring more widely the trade-offs between energy and the SDGs [24]. The consumption of clean energy is limited in rural households, with more than 50% of rural households using kerosene as the primary fuel for lighting; 67% of Rajasthan’s households cook within their dwellings, with a small percentage (6%) who cook in an area that is separate from the living/dwelling buildings and the remainder (27%) of households cooking outdoors [25]. According to data from the National Sample Survey Organization [26], the cooking energy consumption in rural areas is mostly by unprocessed biomass fuels, which accounts for ~85% of total energy consumption, including wood, dung cakes, and crop residues, of which wood is the main cooking fuel that accounts for 77.6%. The average PM2.5 concentrations found in rural and urban kitchens in Rajasthan is estimated to be 20% higher (respectively) than the average level in all Indian households [27]. Household energy consumption patterns vary between rural and urban areas. In urban areas, households mainly use three fuels as primary sources of cooking energy, wood, kerosene, and LPG. During the period from 2004/2005 to 2009/2010, use of noncommercial fuels substantially declined due to high-income residents in cities paying more attention to indoor air quality and replacing biomass fuels with high-quality clean fuels such as LPG [28]. Most households choose to use LPG as a cooking fuel possibly due to factors such as cleanliness, higher efficiency, and the Government of India’s Ujjawala Scheme which provides subsidies to households using LPG. In rural households, traditional biomass fuels still predominate in overall energy consumption LPG is not widely used in poor households due to its high initial cost and the risk of explosions [18,19]. Those poor households will be the focus of our study.

National surveys such as the India National Family Health Survey at household level have examined type of cooking fuel (40% of households use clean fuel for cooking) and exposure to cook fuel smoke in the home, where potential harmful health effects are identified [29]. Rajasthan, compared to other regions in North India, has a large share of households (after Punjab) using solid fuel for cooking as shown in Table 1 below.

A study in Rajasthan covering 1989 households across 13 villages examined the interlinkages between socioeconomic conditions and fuel use and found that the health impacts in the form of respiratory symptoms related to the use of biofuels were higher for adult women [30]. However, there is a gap in the literature on behavioral aspects that brings both the local and regional level contexts together, where adoption and sustained use must look at factors spanning households, communities, societal, and program levels [31] In Rajasthan, there is limited evidence for the association between biofuels and health outcomes but there is not a model established at a regional level to demonstrate the linkages between biomass and health outcomes. There are also not many studies that integrate evidence from different study designs here (quantitative, qualitative, and case study) which can contribute to causal relationships between enabling/inhibiting factors [32]. Therefore, this paper will derive a model for the regional level data examining the relationship between respiratory symptoms and fuel type and to what extent at a district level there may be evidence for causal inferences and/or strong associations.

### 1.3. Study Objectives

There is a strong association established at the global level between women’s health outcomes and biomass cooking fuel in the Indian context and beyond as well as a global dominance of wood consumption; however, there is a need for an evidence base for these claims in context-specific settings at regional and district levels to enable successful application of schemes such as the Ujjawala scheme. The study presents the association between biofuels and health outcomes in Rajasthan, highlighting the lack of region-specific studies and underexplored factors that influence fuel choice from a behavioral perspective. The paper employs a mixed-methods approach using a rapid assessment conducted through household surveys in eight districts in Jaipur to assess behavioral factors and regression modeling of regional data (sample size 41,965 women) from the Demographic and Health Survey (DHS) 7 (2015–2016). The results from the household survey suggest women who cook with biofuels suffer adversely, educational level and age have an impact on fuel consumption, and low household income determines levels of biofuel consumption and clean fuel purchased. The linear regression model provided similar and significant results suggesting a strong association between fuel type and symptoms of respiratory infection accounting for age and education.

## 2. Materials and Methods 

### 2.1. Survey Overview

The DHS survey information included in this analysis comprises individual-level data on women, examining demographic variables such as age and educational attainment. Age was standardized to the categories 26–30, 21–30, 40–49, and women 50+ years for consistency with the Medical Research Council (MRC) survey [33] and to ensure consistency with the rapid household survey which was based on MRC survey template. The number of women meeting the age criteria was 41,965, forming the basis of our analysis. For household information, wealth quintile is considered bearing in mind that there is a strong evidence base for colinearity between wealth quintile and fuel choice [34]. Behavioral and health profile information included cooking fuel, place of cooking, type of fire/stove among households with solid fuels, percentage using clean fuel/solid fuel for cooking, and exposure to smoke inside the home. Health outcome data explored is narrowed to symptoms of an acute respiratory infection such as cough, short rapid breathing that is chest related, and/or difficulty breathing that is chest related. 

### 2.2. Sampling

As the analysis was conducted over a two-stage sampling frame used by the DHS, a complex sample frame was created applying the sample weight (V005) taking into account the clustering and stratification in the sample frame using the ‘Complex Sample’ function in SPSS. This ‘Complex Sampling’ frame takes into account the strata for sampling errors (V022), primary sampling unit (V021), and the weight generated V005/1,000,000 as directed for DHS samples. A subset of the sample only exploring data for Rajasthan was then used including a sample of 41,965 women in our study.

### 2.3. Linkages and Associations to Explore 

To explore linkages between type of cooking fuel and incidence of respiratory symptoms, dummy variables were created drawing upon the data on fuel type and symptoms of cough. With these binary and categorical variables, a general linear regression was conducted using the normalized variables (z-scores) for both the independent (fuel, educational attainment, wealth index, and age) and the dependent variable (cough, problems with the chest and short rapid breaths). Linear regression was considered as a starting point for the analysis as initial cross-tabulations suggested associations. Further consideration of multiple variables also suggested using a multivariate linear regression, which is consistent with studies examining this relationship in other contexts. Other methods and/or more complex modeling approaches such as logistic regression and ANOVA were considered, though the literature review suggested using the evidence of the linear regression as a basis for further investigation.

The ‘type of cooking fuel’ (V161 in the individual records survey for women in the DHS) which included several categories (electricity, LPG, biogas, kerosene, coal, charcoal, wood, straw, agricultural waste, animal, no food, and other) was simplified to a binary variable coded ‘fuel’ which referred to clean fuels (electricity and LPG coded as 1) and everything else (coded as 0). For respiratory symptoms, the models look at the outcome for cough, a dummy variable (yes or no) derived from the ‘cough in the last two weeks’ variable (H31$1 in the DHS) which includes responses ranging from no, yes in the last 24 hours, yes in the last two weeks, don’t know. Dummy variable for short breaths was created from variable H31$1 which included no, yes, yes—last two weeks, and 8—do not know to reported incidence of short breaths (Yes or No). A dummy variable was also created for problems in chest, blocked or running nose (chest only, nose only, both, other, do not know) to reported chest and/or blocked or running nose (Yes/No).

### 2.4. District Level Survey

Based on the literature and the gaps, this study included a rapid small-scale assessment using household survey questionnaires applied for 8 districts in Jaipur. The rapid household assessment enabled us to assess barriers and opportunities for use of clean fuels and perceived health outcomes for a small sample to supplement and complement the evidence derived from the DHS data. Vice versa, the DHS data also enabled us to assess whether our rapid assessment from the survey was consistent with wider regional trends. The health component of the household questionnaire included questions related to the severity of respiratory symptoms was adapted from a Medical Research Council (MRC) questionnaire [33]. 

At the time we started the survey, the Pradhan Mantri Ujjawala Yojana (PMUY) (or in English Prime Minister Ujjawala Scheme) was already launched and in action. 

With respect to bias, response and recall bias was a concern. For response bias, the researchers included measures such as avoiding leading questions, creating an environment consistent with the MRC protocol to encourage participants to respond with information to the best of their knowledge. In order to mitigate the risk of recall bias, the researchers adapted questions using the MRC questionnaire, which has been standardized in terms of language and has been applied to varied contexts and settings. Lastly, to triangulate these findings, the DHS survey results which cover a larger sample are also used to confer findings. 

**Figure 2:**
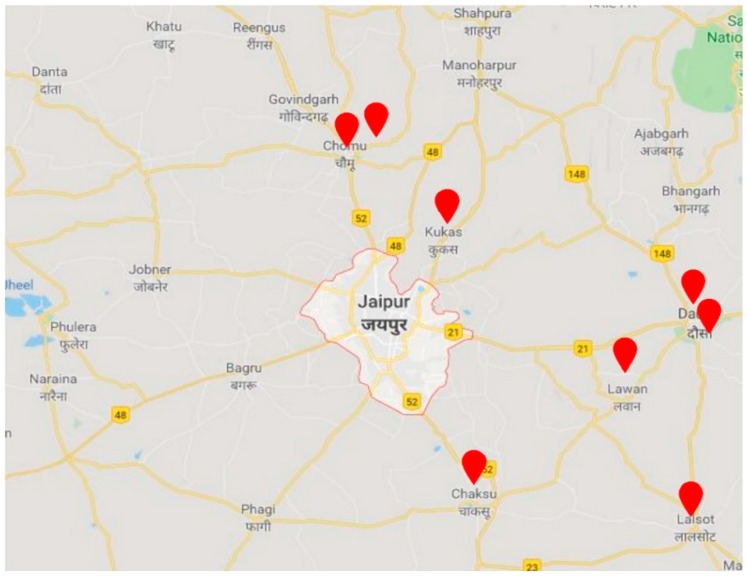
Location of the survey and questionnaire in Rajasthan.

**Figure 3:**
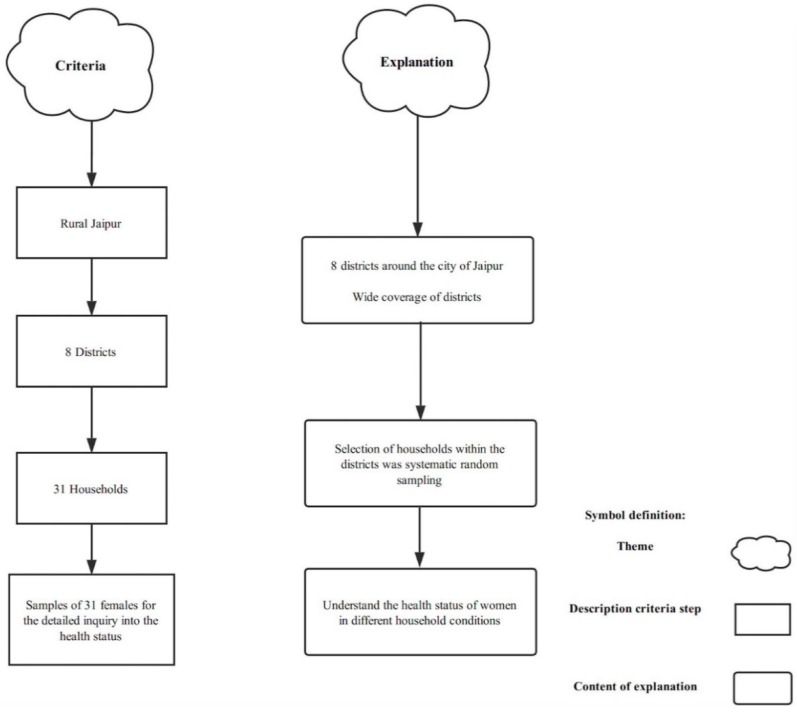
Sampling and approach to household selection.

Using simple random sampling, women from 31 households in 8 districts were selected for participation (women using biofuels, 15 and older and responsible for the collection of wood and indoor cooking) across Jaipur (Figure 2). The inclusion criteria included (a) households who used wood as the cooking fuel (b) respondents were women who were aged 15 or older, (c) women who were responsible for the collection of wood, and (d) women who carried out indoor cooking. See Figure 3 for the approach to sampling. The survey was conducted during July 2018. The questionnaire was designed in English and then translated into Hindi to enable respondents to better understand the content of the study. The in-county household survey was led by Malaviya National Institute of Technology which also ensured accuracy during the fieldwork and analysis of results.

The items in the survey were divided into two categories, individual and household data. The individual information collected included essential, behavioral, and health profile information. Essential information included age, occupation, and education level. Behavioral and health profile information comprised of cooking frequency, cooking duration, cooking methods, and respiratory and nonrespiratory symptoms (Supplementary). Age was disaggregated by the following age groups: 16–20, 21–30, 31–40, and 40 years and above. Occupation was identified by profession and educational level was disaggregated by primary level, junior high school, senior high school, and university education. The survey also included collecting data about the exposure of children to cooking fuel emissions while their mothers were cooking, including the number, gender, and age of children exposed, but did not examine the health status of children. The questions were restricted to respiratory symptoms including coughing, breathlessness, and wheezing. Moreover, women’s smoking habits, religious faith, and types of food cooked were not within the scope of the survey.

The household information gathered principally related to cooking fuel consumption patterns, housing characteristics, and the willingness of respondents to reduce indoor air pollution. The investigation of cooking fuel consumption patterns involved the distribution, time, and effort of procurement/collection of cooking fuel and the health risks experienced during the process of wood procurement/collection. Household characteristics also included monthly household income, monthly cooking fuel costs, cooking fuel consumption, and types of cooking stoves. Though beyond the scope of this paper, an assessment of respondents’ willingness to reduce indoor air pollution also explored willingness to pay and capacity to implement new technologies. House types and sizes, indoor air pollutant concentrations, and kitchen ventilation conditions were excluded from the scope of the rapid household survey.

## 3. Results

### 3.1. Demographic and Health Survey (DHS) Results 

#### Descriptive for the Sample

**Figure 4:**
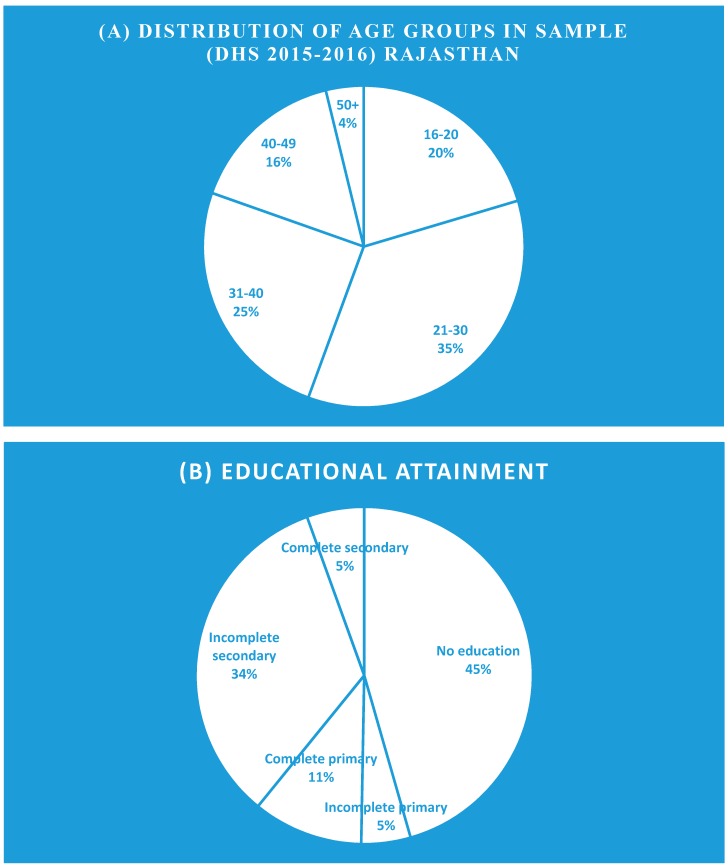
(**A**) Distribution of age group and (**B**) socioeconomic–educational attainment composition of the sample.

Total population for the sample included 41,965 women from the state of Rajasthan (standard error, 0.006) aged 15 and above. The age distribution is shown in Figure 4. In exploring the association between fuel type and respiratory symptoms (cough), educational level and age were taken into account, with the capability to buy clean fuel [34]. With education and age accounted for, the general linear regression yielded the following results on subpopulation (41,965 women) sampled in the state of Rajasthan. The first of four regression models examined linkages between fuel type and cough (as a respiratory symptom).

The R-square variable suggests that 25% of the variance in the dependent variable is explained by the independent variables. In cases where there is a greater amount of unexplainable variation, R-square values can be lower. There may be other reasons related to human behaviour that will not capture these figures. Nonetheless, as all the *p*-values are significant, important conclusions may be drawn about the relationships between the variables. The significance of the coefficients also suggests that there is a mean change in the dependent variable (respiratory systems) given a shift in the independent variable (fuel type) (Table 2).

The association was significant between the fuel type accounting for educational attainment and the age groups, with type. Among the poorest wealth indices, the association is statistically significant. All categories of educational attainment and all age groups yielded statistically significant results with the Wald test whose value parameters were not 0. It confirms that it should be included in the model.

### 3.2. Household Questionnaires 

#### 3.2.1. Socioeconomic Characteristics

Of this subsample of 31 households, all of the respondents reported level of education: 54.8% to primary level, a drop from junior to high school education (19.4% with junior high school and 16.1% for senior high school), and 9.7% attained university education. Among all respondents, 46.7% of respondents were between 21 and 30 years, 23.3% were between the age of 31–40 years, and 30% were over 40 years. However, there were no respondents aged between 15 and 20 years (Figure 4). In terms of occupations, 87% were housewives, 6% were farmers, and 3% were teachers, and 3% of respondents refused to disclose their occupations. For this investigation, the larger proportions of the population were homemakers with primary education at best and aged 21–30 years. 

#### 3.2.2. Cooking Fuel Consumption Patterns

Almost all the households (97%) in rural Jaipur used biomass fuel for cooking, of which 93.5% utilized wood and the remaining 6.5% used crop residues.

#### 3.2.3. Cooking Fuel vs. Prevalence of Respiratory Symptoms

Among respiratory symptoms, the numbers of respondents with coughing and breathing difficulties were observed to be much higher than those with wheezing. All respondents who reported wood and LPG use self-reported their breathing difficulties. Approximately 70% of respondents with coughing symptoms used wood, of which 54% of respondents had a coughing frequency of no more than 50% of days, while 42.4% of respondents have had a cough lasting longer than one month (Table 3). All respondents who have had a cough lasting more than three months were from households that used wood exclusively. Breathing difficulties were the most common respiratory symptoms in surveyed districts regardless of whether households utilized clean fuel or wood. 

#### 3.2.4. Cooking Fuel vs. Socioeconomic Characteristics

Respondents over the age of 30 years generally used wood as their main cooking energy source. Respondents who used LPG were also distributed equally across the three age groups, with respondents who used LPG as their primary cooking energy source mainly concentrated between the ages of 21 and 30 years. The educational levels of respondents play an essential role in the choice of cooking fuels. Mostly, primary and junior high school level educated respondents used a higher proportion of wood and crop residues in the cooking process, with 76% of respondents using wood as their primary cooking fuel. Respondents with senior high school education level or above usually used LPG as the primary fuel, although the occasional use of wood in the cooking process was universal among respondents.

## 4. Discussion

This study combines evidence from a rapid perception study at local level to data captured at regional level (DHS data). The rapid assessment helps to draw out contextual and behavioral aspects which complements the quantitative data captured at regional level. At the regional level, prevalence of respiratory symptoms was highly correlated with use of biofuels as opposed to other clean fuels (kerosene, electricity, and LPG). The differences in age and education yielded significant results (*p* < 0.05) with lower educated women likely to use biofuels. 

Using fuel as a proxy for income, even when accounting for income, lower educational level was more strongly associated with biofuel use in both the survey and the DHS analysis. These differences are consistent with studies examining the relationship between biofuels and adverse health outcomes [35]. By focusing on symptoms rather than specific diseases such as COPD, asthma, or acute respiratory infection, the study broadens the inclusion of symptoms that adversely affect well-being, rather than prevalence of a specific disease. This focus on respiratory symptoms has been explored before, however, not at the local level in recent years and not with a regional perspective as a basis for comparison [3,15]. Applying this localized view with regional-level data (2015–2016) provides an opportunity for further exploration into drivers and possible solutions that may affect a wider scope. 

The results also highlight that women’s education levels have a crucial impact on fuel selection. Within the DHS survey data for Rajasthan, women with education levels of complete secondary education and above tend to use clean fuels in preference to wood, which indicates that women’s educational status positively affects the choice of cooking fuels. This is consistent with findings from Randomised Controlled Trials conducted in two states in India where the level of education was linked to use of clean fuel for cooking [19]. Within the in-depth survey, older women with primary level educations consumed more wood than young women with higher education level. While this is not a basis for demonstrating causality, further investigation could ask what descriptors associated with age could explain why older women consume more wood. This may be related to path dependency, availability, or a range of other factors. 

Household income is a critical factor affecting the consumption patterns of cooking fuels. High-income households usually spend more money on purchasing LPG as an alternative to a portion of wood consumption, while low-income households use more wood and crop residues. This is consistent with the results obtained by Pandey and Chaubal where they found that households with higher incomes used a smaller proportion of biomass fuel [36]. 

The links between multiple socioeconomic variables and wood consumption indicate that wood consumption could be reduced by raising female education levels and by increasing household income and reducing the number of meals cooked. Meanwhile, these factors could significantly reduce the exposure of women to health risks caused by using wood. As children are often in close proximity to mothers, further research should incorporate this dimension as it was beyond the scope of this study. Improving women’s education levels would substantially increase the likelihood of households using clean fuel that would require analysis in order to assess impact on the wider society.

## 5. Conclusions

The paper demonstrates the need for sustained and targeted efforts to enhance access to clean and affordable energy sources for women to address barriers to health and well-being, such as symptoms of acute respiratory infection, using a case from Rajasthan. Women from lower socioeconomic backgrounds suffer adversely and are disadvantaged as they spend time collecting fuel which reduces their ability to engage in other productive activities. The strengths of this study are that it approaches this issue from a regional and district level employing mixed methods (regression and survey analysis) to identify strong associations and nuances according to education and age. Where this study could improve is in the system’s level interactions between women’s exposure and children’s exposure as well as include data related to atmospheric data (PM2.5, PM10, NO, and SO_2_) in conjunction with the seasons or monthly temperature as these risk factors are associated with respiratory symptoms. Other potential confounders that would need to be explored more include factors related to social, socioeconomic status such as education, technology access, and cultural differences that may predispose one group to certain behaviors over others. The authors are aware that collinearity and/or other confounders could distort results. Nonetheless, this was a rapid assessment coupled with a wider DHS survey to set out future research in this domain. Other confounders such as environmental tobacco, for which there was no data in the DHS, should also be considered as it may confound the relationship between respiratory symptoms and the variables described here. 

Further research should explore these health linkages and their impact on children taking the diversity of the previous factors into account and including more specifically diagnosed outcomes. Recommendations include targeting women from lower socioeconomic backgrounds with interventions that improve access to clean fuels and provide health services that address symptoms as well as causes. Recalling that the Government of India’s Pradhan Mantri Ujjawala Yojana (LPG Scheme) aims to provide LPG connections to women from below poverty line, this paper provides an evidence base at regional and district level to inform and support schemes like Ujjawala which have the mandate of improving access to clean cooking fuels.

## Figures and Tables

**Table 1:** Percentage of households with electricity and using solid fuel for cooking by state/union territory [29].

State/Union Territory	With Electricity	Using Solid Fuel for Cooking
Punjab	99.6	92.9
Rajasthan	91.0	67.8
Himachal Pradesh	99.5	62.5
Uttarakhand	97.5	48.1
Haryana	98.8	47.4
Delhi	99.8	1.6
Jammu and Kashmir	97.4	41.5
Chandigarh	99.6	4.1

This table is adapted based on the original in the India National Family Health Survey i.e. DHS (Demographic and Health Survey) [29].

**Table 2:** Fuel type vs. respiratory symptoms (cough) vs. tests of model effects.

Source	df1	df2	Wald F	Sig.
Model	12.000	1491.000	892.557	0.001
(Intercept)	1.000	1502.000	290.149	0.001
Fuel (Z-score)	1.000	1502.000	18.621	0.001
Edu (Z-score)	5.000	1498.000	54.716	0.001
Age (Z-score)	6.000	1497.000	1659.738	0.001
R Square	0.253			
Cough = (Intercept) + Fuel + Education + Age				

**Table 3:** Coughing in respondents (N = Number of respondents; Numbers in parentheses indicate percentage of respondents).

Disease Symptoms		Cooking Fuels			
		All (N = 26)	Clean Fuel (N = 6)	Wood (N = 18)	Crop Residues (N = 2)
**Duration time**	Less than 5 days	3 (11.5%)	0	3 (11.5%)	0
	5–10 days	3 (11.5%)	1(3.8%)	1 (3.8%)	1 (3.8%)
	10–30 days	6 (23.1%)	2 (7.7%)	3 (11.5%)	1 (3.8%)
	1–3 months	10 (38.5%)	3 (11.5%)	7 (27.0%)	0
	3–6 months	4 (15.4%)	0	4 (15.4%)	0
**Cough in one day (%)**	Less than 25%	15 (57.7%)	4 (15.4%)	11(42.3%)	0
	25–50%	5 (19.2%)	0	3 (11.5%)	2 (7.7%)
	More than 50%	4 (15.4%)	1 (3.8%)	3 (11.5%)	0
	All day	2 (7.7%)	1 (3.8%)	1 (3.8%)	0

## Supplementary Materials

The following are available online at https://www.mdpi.com/1660-4601/16/19/3594/s1, Table S1 Age Groups, Table S2 Educational Attainment.
