# Supplementary material for: Linkages between Respiratory Symptoms in Women and Biofuel Use: Regional Case Study of Rajasthan, India
Source: Int J Environ Res Public Health. 2019 Sep 25;16(19):3594. doi: 10.3390/ijerph16193594 (PMC6801899; doi:10.3390/ijerph16193594)
Supplement: Supplementary file 1 [file ijerph-16-03594-s001.pdf]

# Supplementary Information

| Age Groups | | | | | |
|---|---|---|---|---|---|
| | | Estimate | Standard Error | 95% Confidence Interval | |
| | | | | Lower | Upper |
| Population Size | 16-20 | 8566.89 | 116.97 | 8337.45 | 8796.34 |
| | 21-30 | 14774.34 | 141.20 | 14497.38 | 15051.31 |
| | 31-40 | 10405.04 | 110.01 | 10189.26 | 10620.82 |
| | 40-49 | 6610.09 | 87.92 | 6437.63 | 6782.54 |
| | 50+ | 1608.64 | 43.43 | 1523.46 | 1693.82 |
| | Total | 41965.00 | 262.94 | 41449.24 | 42480.76 |
| % of Total | 16-20 | 20.4% | 0.2% | 20.0% | 20.8% |
| | 21-30 | 35.2% | 0.3% | 34.7% | 35.7% |
| | 31-40 | 24.8% | 0.2% | 24.3% | 25.3% |
| | 40-49 | 15.8% | 0.2% | 15.4% | 16.1% |
| | 50+ | 3.8% | 0.1% | 3.6% | 4.0% |
| | Total | 100.0% | 1.0% | 100.0% | 100.0% |

| Educational Attainment | | | | | |
|---|---|---|---|---|---|
| | | Estimate | Standard Error | 95% Confidence Interval | |
| | | | | Lower | Upper |
| Population Size | No education | 16993.82 | 189.48 | 16622.14 | 17365.49 |
| | Incomplete primary | 1767.22 | 50.85 | 1667.48 | 1866.95 |
| | Complete primary | 3942.85 | 77.24 | 3791.34 | 4094.36 |
| | Incomplete secondary | 12548.54 | 164.50 | 12225.87 | 12871.21 |
| | Complete secondary | 2075.39 | 54.21 | 1969.05 | 2181.73 |
| | Higher | 4637.20 | 129.63 | 4382.92 | 4891.47 |
| | Total | 41965.00 | 262.94 | 41449.24 | 42480.76 |
| % of Total | No education | 40.5% | 0.4% | 39.7% | 41.3% |
| | Incomplete primary | 4.2% | 0.1% | 4.0% | 4.4% |
| | Complete primary | 9.4% | 0.2% | 9.1% | 9.7% |
| | Incomplete secondary | 29.9% | 0.3% | 29.3% | 30.5% |
| | Complete secondary | 4.9% | 0.1% | 4.7% | 5.2% |
| | Higher | 11.1% | 0.3% | 10.5% | 11.7% |
| | Total | 100.0% | 0.0% | 100.0% | 100.0% |
